# Pharmacologic ATM but not ATR kinase inhibition abrogates p21-dependent G1 arrest and promotes gastrointestinal syndrome after total body irradiation

**DOI:** 10.1038/srep41892

**Published:** 2017-02-01

**Authors:** Frank P. Vendetti, Brian J. Leibowitz, Jennifer Barnes, Sandy Schamus, Brian F. Kiesel, Shira Abberbock, Thomas Conrads, David Andy Clump, Elaine Cadogan, Mark J. O’Connor, Jian Yu, Jan H. Beumer, Christopher J. Bakkenist

**Affiliations:** 1 Department of Radiation Oncology, University of Pittsburgh School of Medicine, Pittsburgh, PA, USA; 2 Department of Pathology, University of Pittsburgh School of Medicine, Pittsburgh, PA, USA; 3 DNA Damage Response Biology Area, Oncology IMED, AstraZeneca, Cambridge, UK; 4 Cancer Therapeutics Program, University of Pittsburgh Cancer Institute, Pittsburgh, PA, USA; 5 Department of Biostatistics, University of Pittsburgh School of Public Health, Pittsburgh, PA, USA; 6 Inova Schar Cancer Institute, Inova Center for Personalized Health, Falls Church, VA, USA; 7 Department of Pharmaceutical Sciences, School of Pharmacy, University of Pittsburgh, Pittsburgh, PA, USA

## Abstract

We show that ATM kinase inhibition using AZ31 prior to 9 or 9.25 Gy total body irradiation (TBI) reduced median time to moribund in mice to 8 days. ATR kinase inhibition using AZD6738 prior to TBI did not reduce median time to moribund. The striking finding associated with ATM inhibition prior to TBI was increased crypt loss within the intestine epithelium. ATM inhibition reduced upregulation of p21, an inhibitor of cyclin-dependent kinases, and blocked G1 arrest after TBI thereby increasing the number of S phase cells in crypts in wild-type but not *Cdkn1a(p21*^*CIP/WAF1*^)*−/−* mice. In contrast, ATR inhibition increased upregulation of p21 after TBI. Thus, ATM activity is essential for p21-dependent arrest while ATR inhibition may potentiate arrest in crypt cells after TBI. Nevertheless, ATM inhibition reduced median time to moribund in *Cdkn1a(p21*^*CIP/WAF1*^)*−/−* mice after TBI. ATM inhibition also increased cell death in crypts at 4 h in *Cdkn1a(p21*^*CIP/WAF1*^)*−/−*, earlier than at 24 h in wild-type mice after TBI. In contrast, ATR inhibition decreased cell death in crypts in *Cdkn1a(p21*^*CIP/WAF1*^)*−/−* mice at 4 h after TBI. We conclude that ATM activity is essential for p21-dependent and p21-independent mechanisms that radioprotect intestinal crypts and that ATM inhibition promotes GI syndrome after TBI.

The rapid and continuous division of labile stem cells to replace the short-lived functionally mature cells in the epidermis, hematopoietic system, and gastrointestinal (GI) tract renders them sensitive to genotoxic stress, and the irreversible loss of these stem cells and their descendants from an organism results in acute radiation disease within days of exposure to ionizing radiation (IR)^1^. The hematopoietic and GI syndromes that follow acute radiation exposure are caused by infection and hemorrhage at 12–20 days, and infection, dehydration, and electrolyte imbalance at 7–12 days, respectively^2^,^3^. Accordingly, dose-limiting toxicity is observed in the radiation oncology clinic in bone marrow and the small intestine and an understanding of mechanisms that protect normal tissues following exposure to IR is essential for the development of improved cancer therapies.

Ataxia telangiectasia-mutated (ATM) and ataxia telangiectasia and Rad3-related (ATR) are serine/threonine protein kinases activated at DNA double-strand breaks (DSBs) and damaged replication forks, respectively^4^,^5^. Since ataxia telangiectasia (A-T) patients who express no ATM protein are the most radiosensitive patients identified, pharmacologic ATM kinase inhibitors may increase the efficacy of targeted radiotherapy^6^,^7^,^8^. Consistent with this premise, ATM inhibitors sensitize cancer cells to IR *in vitro*^9^,^10^,^11^. The outcomes of ATM inhibition *in vivo* are harder to predict, however, as while *Atm−/−* knockout mice are viable^12^,^13^, expression of ATM kinase-inactive in knockin mouse models causes early embryonic lethality^14^,^15^. This suggests that ATM inhibition does not phenocopy ATM protein disruption *in vivo*^16^. Furthermore, ATM protein disruption does not result in the same phenotype in all cells *in vivo*. While the GI tract in *Atm−/−* mice is radiosensitive, the developing nervous system in *Atm−/−* mice is radioresistant^13^. Less is known about the physiological consequences of ATR disruption as the protein is essential in mice and mammalian cells^17^,^18^,^19^,^20^. ATR kinase inhibitors sensitize cancer cells to IR *in vitro*, but are less potent than ATM inhibitors in this respect^21^,^22^,^23^,^24^,^25^,^26^,^27^.

ATM and ATR phosphorylate a broad and overlapping catalogue of several thousand substrates and these modified proteins collectively impact most cellular processes^28^,^29^,^30^. The ATM kinase-dependent phosphorylation and stabilization of p53 after IR may contribute to different phenotypic outcomes observed in different cells^31^,^32^. p53 is required for the upregulation of PUMA which induces Bax/Bak and mitochondrial-dependent apoptosis in hematopoietic stem cells leading to hematopoietic syndrome after irradiation^3^,^31^,^32^,^33^,^34^,^35^. p53 is also required for the upregulation of p21, an inhibitor of several cyclin-dependent kinases (CDKs), which induces G1 cell cycle arrest in intestinal crypt stem cells and this prevents GI syndrome after irradiation^3^,^35^,^36^,^37^,^38^,^39^. Thus, while selective ATM inhibitors may radioprotect the hematopoietic system by blocking the upregulation of p53 and PUMA, ATM inhibitors may concurrently radiosensitize the GI tract by blocking the upregulation of p53 and p21. However, the contribution of ATR and other kinase signaling to p53 activities in these systems is not known, and preclinical animal studies are needed to determine the physiological consequences of ATM and ATR inhibition *in vivo*. Here we explore the physiological consequences of a single dose of first orally active and bioavailable ATM kinase inhibitor, AZ31^40^, or the orally active and bioavailable ATR kinase inhibitor, AZD6738^26^, prior to 9 or 9.25 Gy total body irradiation (TBI).

## Results

### Impact of ATM and ATR kinase inhibition on mouse survival after TBI

**Figure 1:**
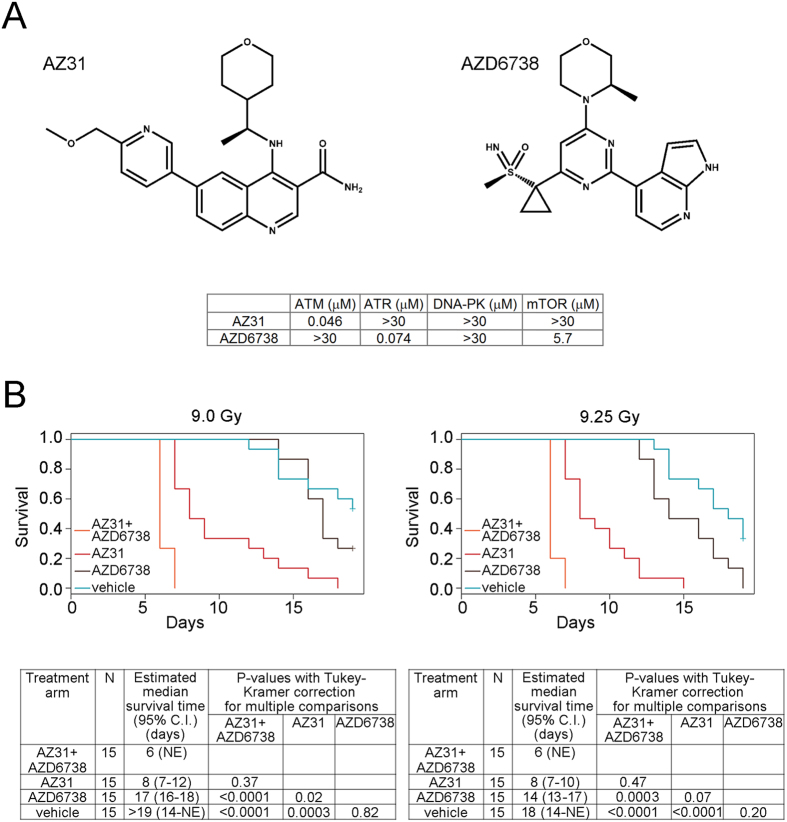
Impact of ATM and ATR kinase inhibition on mouse survival after TBI. (**A**) Chemical structures of the ATM kinase inhibitor, AZ31, and the ATR kinase inhibitor, AZD6738, and selectivity data showing inhibition potency (IC_50_) against ATM, ATR, DNA-PK, and mTOR in cell based assays. (**B**) Kaplan-Meier survival curves for mice treated with a single oral dose of vehicle, 100 mg/kg AZ31, 75 mg/kg AZD6738, or 100 mg/kg AZ31 and 75 mg/kg AZD6738 2 h prior to 9 or 9.25 Gy TBI, and corresponding data tables showing N per cohort, estimated median survival, and adjusted P-values for multiple comparisons among cohorts.

To identify essential ATM and essential ATR kinase signaling, we dosed twelve groups of fifteen female C57BL/6 mice with vehicle, 100 mg/kg AZ31, a selective ATM kinase inhibitor^40^, 75 mg/kg AZD6738, a selective ATR kinase inhibitor^26^, or 100 mg/kg AZ31 + 75 mg/kg AZD6738 2 h prior to 0, 9, or 9.25 Gy TBI (Fig. 1A). These doses of AZ31 and AZD6738 have been described previously and are not associated with known off-target effects^26^,^40^. These are the highest doses that would be used in the clinic. Single agent activity with lower doses of AZD6738 and radiation dose enhancement with lower doses of AZ31 have been observed (data not shown). The doses of TBI were chosen as they were empirically determined to be near the LD50 for C57BL/6 housed in the Hillman Cancer Center Animal Facility and irradiated using a ^137^Cs source (Fig. 1B). Mice were weighed daily and euthanized when moribund, defined as a loss of 20% body weight or a lack of movement and response after being handled. No effect of a single dose of AZ31 and/or AZD6738 was observed in the un-irradiated mice. When the mice received 9 Gy TBI, the cohort treated with both AZ31 and AZD6738 had a shorter overall survival than the cohorts treated with AZD6738 alone (estimated median survival of 6 days vs. 17 days respectively, p < 0.0001) or vehicle (estimated median survival greater than 19 days, p < 0.0001) (Fig. 1B). However, there was no difference in survival detected between the cohort treated with both AZ31 and AZD6738 and the cohort treated with AZ31 alone (estimated median survival of 6 days vs. 8 days, p = 0.37). Among the mice that received 9.25 Gy TBI, the cohort that was treated with both AZ31 and AZD6738 had shorter overall survival compared with the AZD6738 cohort (estimated median survival of 6 days vs. 14 days respectively, p = 0.0003) and the vehicle treated cohort (estimated median survival of 18 days, p < 0.0001). Again, there was no difference in survival detected between the cohort treated with both AZ31 and AZD6738 and the cohort treated with AZ31 alone (estimated median survival of 6 days vs. 8 days, p = 0.47). The time to moribund suggested that ATM inhibition using AZ31 was inducing GI syndrome in mice treated with AZ31 prior to TBI^2^,^3^.

**Figure 2:**
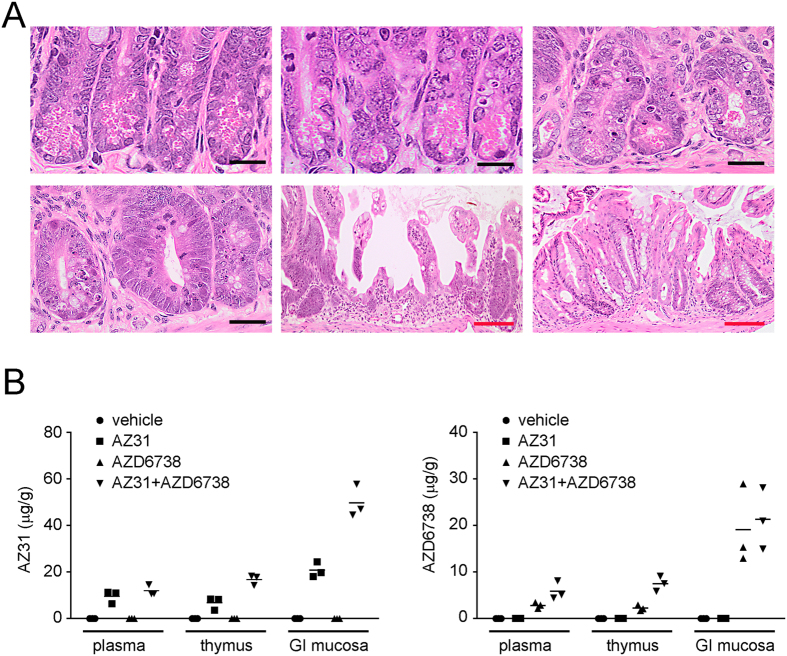
Histopathology associated with AZ31 in moribund mice after TBI and pharmacokinetics of AZ31 and AZD6738 at the time of irradiation. (**A**) Photomicrographs of transverse sections of mouse intestine following 9 Gy TBI. Top left: normal small intestinal crypt epithelium following 9 Gy alone (Day 14). Top center: minimal disruption of morphology within the small intestinal crypt epithelium following 75 mg/kg AZD6738 and 9 Gy (Day 14). Top right: disrupted crypt morphology within the small intestine following 100 mg/kg AZ31 and 9 Gy (Day 7). Bottom left: regenerative crypt hyperplasia within the small intestine following 100 mg/kg AZ31 + 75 mg/kg AZ6738 and 9 Gy (Day 6). Bottom center and bottom right: regions of significant atrophy and loss of the crypt epithelium within the small intestine and large intestine, respectively, following AZ31 + AZ6738 and 9 Gy (Day 6). Black bar = 20 μm; red bar = 100 μm. (**B**) Concentrations of AZ31 and AZD6738 in the plasma, thymus, and small intestinal (GI) mucosa of mice 2 h following a single oral dose of vehicle, 100 mg/kg AZ31, 75 mg/kg AZD6738, or 100 mg/kg AZ31 + 75 mg/kg AZD6738. N = 3 mice per treatment group.

Brain, colon, esophagus, femur, heart, kidney, liver, lung, small intestine, spleen, sternum and stomach were harvested from four mice in each of the treatment groups when they became moribund. Histopathological findings were generally related to the TBI and included hypocellularity of the sternal and femoral bone marrow and the splenic white pulp areas. The striking finding associated with either ATM or ATR inhibition was significant crypt loss within the large and small intestine epithelium in the mice treated with AZ31 and TBI (Fig. 2A). Degeneration of the small and large intestinal crypt epithelium was observed in mice treated with either AZ31 or the combination of AZ31 and AZD6738 prior to TBI. In addition, randomly distributed multifocal atrophy and/or complete loss of the crypt epithelium was evident in the small and large intestine of mice treated with both AZ31 and AZD6738 (and to a lesser extent AZ31 alone) prior to TBI. Within affected regions, there was attenuation of the superficial epithelium with cellular and nuclear pleomorphism/cytomegaly, variable vacuolation, and occasional exfoliation of the epithelium. Atrophic crypts were flanked by tortuous hyperplastic crypts with a distinct basophilic appearance and obvious nuclear crowding, consistent with regenerative hyperplasia in these areas. Conversely, in mice treated with AZD6738 prior to TBI, there was minor degeneration within the crypts but no evidence of crypt atrophy.

### Pharmacokinetics of AZ31 and AZD6738

We assessed exposure of AZ31 and AZD6738 in plasma, thymus, and small intestinal (GI) mucosa 2 h after oral dosing with vehicle, 100 mg/kg AZ31, 75 mg/kg AZD6738, or 100 mg/kg AZ31 and 75 mg/kg AZD6738 (vehicles were mixed 1:1). Co-administration of AZ31 with AZD6738 resulted in a 2.5-fold increase in AZ31 concentrations in thymus and GI mucosa, while the co-administration of AZD6738 with AZ31 caused an approximate 2-3-fold increase in exposure of the AZD6738 in plasma and thymus (Fig. 2B). Due to this apparent interaction between AZ31 and AZD6738 at this timepoint *in vivo*, which may confound the interpretation of downstream pharmacodynamic results, no further experiments were undertaken with the drug combination.

### Impact of ATM and ATR kinase inhibition on intestinal crypt survival after TBI

As the time to moribund mouse is different for each treatment group, we examined the small intestine at standard timepoints after TBI. Labile stem cells in the intestinal crypts divide rapidly to generate cells that migrate up the villi and differentiate into short-lived functionally mature cells that shed from the villi by the normal course of events^41^. Since the proliferating early progenitor and stem cells in intestinal crypts are more sensitive to radiation than the differentiated cells on the villi, crypt degeneration is evident within hours of IR and in the absence of pathology in the villi. Within days of IR, the villi shorten and shrink as replacement cells are not generated in the depopulated crypts^3^,^41^. Stem cell loss occurs with doses of IR above 12 Gy, while doses of approximately 15 Gy result in sterilization of the majority of crypts^42^,^43^.

**Figure 3:**
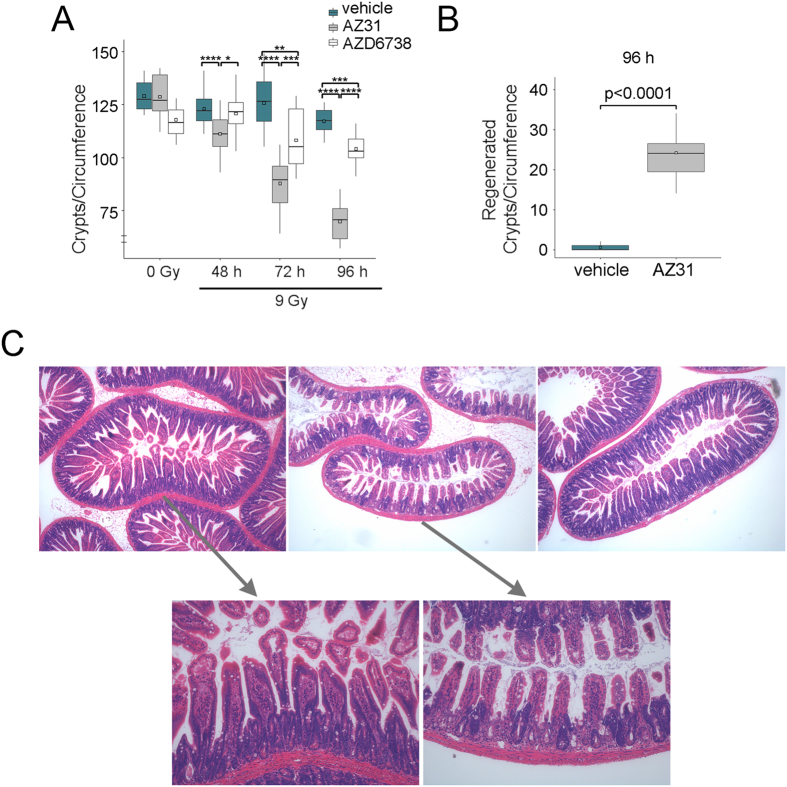
Impact of ATM and ATR kinase inhibition on intestinal crypt survival after TBI. Mice received vehicle, 100 mg/kg AZ31, or 75 mg/kg AZD6738 2 h prior to 9 Gy TBI. Small intestine tissues were harvested at the specified timepoints after TBI. For un-irradiated mice, tissues were harvested at 6 h after inhibitor dosing, equivalent to 4 h after TBI. (**A**) Enumeration of the number of crypts per intact circumference of small intestine at 48 h, 72 h, and 96 h after 9 Gy TBI, compared to 0 Gy controls. Box and whisker plots depict 10–14 total circumferences (n = 10–14), with 5–8 circumferences from each of 2 mice. (**B**) Enumeration of the number of regenerated crypts per circumference of small intestine in irradiated mice at 96 h after 9 Gy TBI. Box and whisker plots depict 15 total circumferences (n = 15), with 7–8 circumferences from each of 2 mice. *p < 0.05, **p < 0.01, ***p < 0.001, ****p < 0.0001. (**C**) Photomicrographs of transverse sections of mouse intestine at 96 h after 9 Gy TBI. Top images (40x magnification), from left to right: vehicle, AZ31, AZD6738. Bottom images represent zoomed sections of vehicle (left) and AZ31 (right) small intestine.

To assess crypt loss after radiation, we enumerated crypts per intact small intestine circumference in irradiated mice at 48 h, 72 h and 96 h after 9 Gy TBI, as well as in un-irradiated mice at 6 h after dosing (Fig. 3A)^35^. ATM inhibition prior to TBI promoted crypt loss by 48 h (mean 123 for vehicle vs. 111 for AZ31, p < 0.0001). Further reductions in crypt number were observed at 72 h (mean 126 for vehicle vs. 87.9 for AZ31, p < 0.0001) and 96 h (mean 117 for vehicle vs. 69.9 for AZ31, p < 0.0001). ATR inhibition prior to TBI also decreased crypt number, to a lesser degree, at 72 h (mean 126 for vehicle vs. 108 for AZD6738, p = 0.003) and 96 h (mean 117 for vehicle vs. 104 for AZD6738, p = 0.0003). Expectedly, only minor crypt loss was observed by 96 h after TBI alone (mean 129 for un-irradiated vehicle control vs. 117 for irradiated vehicle), as 9 Gy is insufficient to irreparably damage the stem cell compartment^42^,^44^.

Three to four days after treatments that induce significant but non-lethal injury to intestinal crypt stem cells, regenerative hyperplasia is seen as these cells repopulate the damaged crypt epithelium^41^,^43^. ATM kinase inhibition increased the number of regenerated crypts at 96 h after TBI (mean 0.53 for vehicle vs. 24.2 for AZ31, p < 0.0001) (Fig. 3B). ATM kinase inhibition caused both crypt regeneration and crypt loss at 96 h after TBI (Fig. 3C). The absence of regenerated crypts in the vehicle treated mice after TBI suggests that the injury was insufficient to induce a regenerative response in the intestinal crypt stem cells.

**Figure 4:**
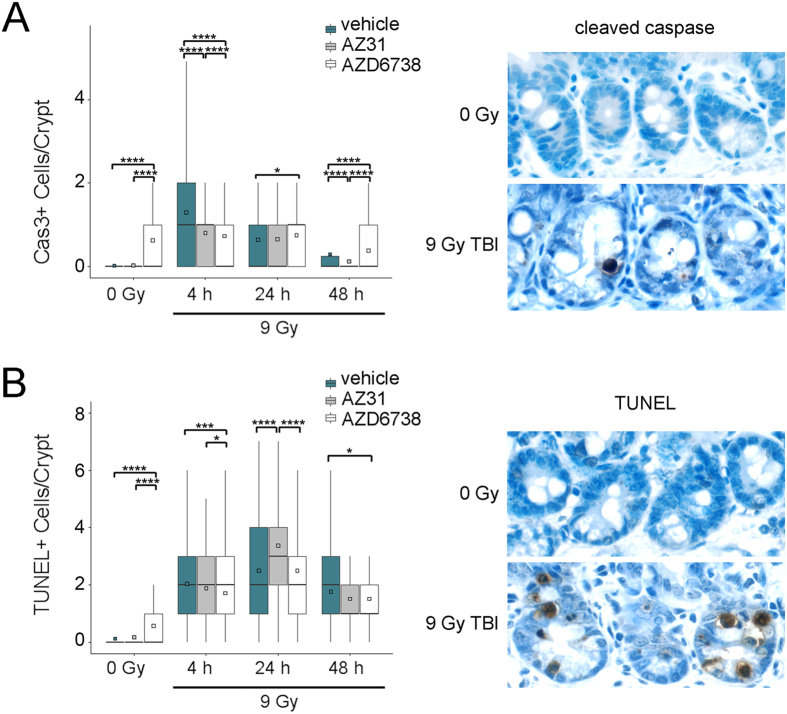
Impact of ATM and ATR kinase inhibition on intestinal crypt cell death after TBI. Mice received vehicle, 100 mg/kg AZ31, or 75 mg/kg AZD6738 2 h prior to 9 Gy. Small intestine tissues were harvested at the specified timepoints after TBI. For un-irradiated control mice, tissues were harvested at 6 h after inhibitor dosing, equivalent to 4 h after TBI. (**A**) Left: Enumeration of the number of cleaved caspase (Cas3) positive cells per small intestine crypt in vehicle, AZ31, or AZD6738 treated mice at 4 h, 24 h, and 48 h after 9 Gy TBI, compared to 0 Gy controls. Right: Representative images of cleaved caspase positive IHC staining in the small intestine crypts of vehicle treated mice at 24 h after 9 Gy TBI, compared to 0 Gy control. (**B**) Left: Enumeration of the number of TUNEL positive cells per small intestine crypt in vehicle, AZ31, or AZD6738 treated mice at 4 h, 24 h, and 48 h after 9 Gy TBI, compared to 0 Gy controls. Right: Representative images of TUNEL positive IHC staining in the small intestine crypts of vehicle treated mice at 24 h after 9 Gy TBI, compared to 0 Gy control. For Cas3 and TUNEL quantitation, box and whisker plots depict counts from a total of 200 crypts (n = 200), with 100 crypts from each of 2 mice. *p < 0.05, ***p < 0.001, ****p < 0.0001.

To determine the extent of cell death in intestinal crypts we performed immunohistochemistry for cleaved caspase 3, indicative of apoptosis, and the TUNEL assay for fragmented DNA and enumerated the number of positive cells per crypt. AZD6738 induced apoptosis in the intestinal crypts of un-irradiated mice (mean 0.63 for AZD6738 vs. 0.02 for vehicle, p < 0.0001) (Fig. 4A). At 4 h after 9 Gy TBI, equivalent to the timepoint of the un-irradiated controls, the number of apoptotic cells per crypt was reduced by either ATM or ATR inhibition prior to TBI compared to TBI alone (mean 1.30 for vehicle vs. 0.81 for AZ31, p < 0.0001; mean 1.30 for vehicle vs. 0.73 for AZD6738, p < 0.0001). At 24 h and 48 h after TBI, apoptosis remained elevated in crypts of mice treated with AZD6738 compared to those of mice treated with vehicle (mean 0.64 for vehicle vs. 0.76 for AZD6738 at 24 h, p = 0.04; mean 0.30 for vehicle vs. 0.39 for AZD6738 at 48 h, p < 0.0001). Conversely, the number of apoptotic crypts cells in AZ31 treated and vehicle treated mice were similar at 24 h after TBI (mean 0.64 for vehicle vs 0.68 for AZ31, p = 0.64), but fewer apoptotic cells were present in the crypts of AZ31 treated mice at 48 h after TBI (mean 0.30 for vehicle vs 0.13 for AZ31, p < 0.0001).

Treatment with AZD6738 induced fragmented DNA in intestinal crypts in the un-irradiated mice (mean 0.58 for AZD6738 vs 0.13 for vehicle, p < 0.0001) (Fig. 4B). At 24 h after 9 Gy TBI, the number of cells containing fragmented DNA was greatest in the AZ31 treatment group (mean 2.50 for vehicle vs. 3.37 for AZ31, p < 0.0001). At 4 h and 48 h after TBI, the number of cells per crypt containing fragmented DNA was greatest in the vehicle groups, and this radiation-induced increase was reduced at both timepoints by ATR inhibition (mean 2.05 for vehicle vs. 1.72 for AZD6738 at 4 h, p = 0.0005; mean 1.77 for vehicle vs. 1.56 for AZD6738 at 48 h, p = 0.02).

### Impact of ATM and ATR kinase inhibition on DNA synthesis in intestinal crypts after TBI

**Figure 5:**
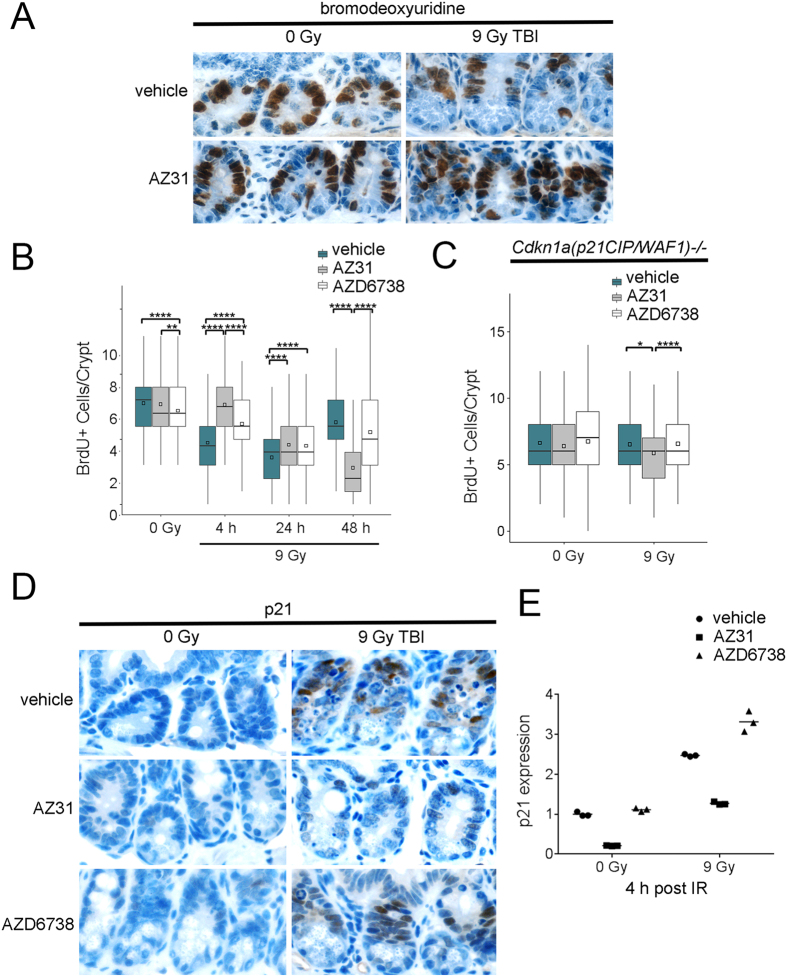
Impact of ATM and ATR kinase inhibition on DNA synthesis in intestinal crypts after TBI. Mice received vehicle, 100 mg/kg AZ31, or 75 mg/kg AZD6738 2 h prior to 9 Gy TBI. Small intestine tissues were harvested at the specified timepoints after TBI. For un-irradiated mice, tissues were harvested at 6 h after inhibitor dosing, equivalent to 4 h after TBI. (**A**) Representative images of bromodeoxyuridine (BrdU) positive IHC staining in the small intestine crypts of vehicle and AZ31 treated, wild-type mice at 4 h after 9 Gy TBI, compared to 0 Gy controls. (**B**) Enumeration of the number of BrdU positive cells per small intestine crypt in vehicle, AZ31, or AZD6738 treated, wild-type mice at 4 h, 24 h, and 48 h after 9 Gy TBI, compared to 0 Gy controls. (**C**) Enumeration of the number of BrdU positive cells per small intestine crypt in vehicle, AZ31, or AZD6738 treated, *Cdkn1a(p21*^*CIP/WAF1*^)*−/−* mice at 4 h after 9 Gy TBI, compared to 0 Gy controls. For BrdU quantitation in both wild-type and *Cdkn1a(p21*^*CIP/WAF1*^)*−/−* mice, box and whisker plots depict counts from a total of 200 crypts (n = 200), with 100 crypts from each of 2 mice. *p < 0.05, **p < 0.01, ****p < 0.0001. (**D**) Representative images of p21 positive IHC staining in the small intestine crypts of vehicle, AZ31, or AZD6738 treated, wild-type mice at 4 h after 9 Gy TBI, compared to 0 Gy controls. (**E**) p21 mRNA expression in the small intestine mucosa of vehicle, AZ31, or AZD6738 treated, wild-type mice at 4 h after 9 Gy TBI, compared to 0 Gy controls. Plots depict 3 technical replicates from one mouse per condition.

p53 is required for the upregulation of p21 which induces G1 cell cycle arrest in intestinal crypt stem cells and this prevents GI syndrome after irradiation^3^,^35^,^36^,^37^,^38^,^39^. To determine whether AZ31 and AZD6738 impact the number of crypt cells in S phase, we treated mice with vehicle, 100 mg/kg AZ31, or 75 mg/kg AZD6738 2 h prior to 0 Gy or 9 Gy TBI and labelled DNA synthesis *in vivo* by injecting BrdU into the intraperitoneal cavity 2 h prior to euthanasia. Incorporated BrdU was detected by immunohistochemistry and the number of S phase cells per crypt was enumerated for each treatment group (Fig. 5A). Treatment with AZD6738 reduced the number of cells in S phase in the intestinal crypts of un-irradiated control animals (mean 7.21 for AZD6738 vs. 7.79 for vehicle, p < 0.0001) (Fig. 5B). At 4 h after TBI, equivalent to the timepoint of the un-irradiated controls, the number of S phase cells in vehicle treated mice was reduced by 39% compared to un-irradiated vehicle control (mean 4.75 for TBI vs 7.79 for no IR, p < 0.0001), and this reduction was not evident in the AZ31 treated animals (mean 7.65 for AZ31 vs. 4.75 for vehicle, p < 0.0001). In AZD6738 treated mice, the number of S phase cells at 4 h after TBI was also reduced, but to a lesser extent than in the vehicle treated animals (mean 6.19 for AZD6738 vs. 4.75 for vehicle, p < 0.0001). In both vehicle and AZD6738 treated mice, BrdU incorporation was further reduced at 24 h, but increased to similar levels by 48 h after TBI (mean 6.31 for vehicle vs. 5.61 for AZD6738, p = 0.14). Conversely, from 4 h to 48 h after TBI, AZ31 treated mice exhibited a progressive decline in the number of intestinal crypt cells in S phase (mean 2.83 for AZ31 vs. 6.31 for vehicle, p < 0.0001; 2.83 for AZ31 vs. 5.61 for AZD6738, p < 0.0001).

ATM is required for p53 stabilization^31^ and p21 upregulation in cells cultured *in vitro*^36^,^37^. To determine whether ATM and ATR inhibition prior to TBI impacts the number of cells in S phase in the absence of the p21-dependent G1 cell cycle checkpoint, we treated *Cdkn1a(p21*^*CIP/WAF1*^)*−/−* mice with vehicle, 100 mg/kg AZ31, or 75 mg/kg AZD6738 2 h prior to 0 Gy or 9 Gy TBI and labelled DNA synthesis *in vivo* with BrdU. Treatment with AZ31 prior to TBI reduced the number of S phase cells per crypt at 4 h after TBI compared to treatment with vehicle or AZD6738 (mean 6.52 for vehicle vs. 5.86 for AZ31, p = 0.04; mean 6.57 for AZD6738 vs. 5.86 for AZ31, p < 0.0001) (Fig. 5C). However, this reduction represented only an 8.4% decrease relative to mean number of S phase cells per crypt in un-irradiated mice treated with AZ31.

To confirm that ATM inhibition prior to TBI ablates the radiation-induced, p21-dependent G1 cell cycle checkpoint, we treated wild-type mice with vehicle, 100 mg/kg AZ31, or 75 mg/kg AZD6738 2 h prior to 0 Gy or 9 Gy TBI and examined p21 protein expression in the intestinal crypts at 4 h after TBI. While p21 protein was clearly evident in intestinal crypt cells of irradiated mice treated with vehicle or AZD6738, AZ31 blocked radiation-induced p21 expression (Fig. 5D). In addition, we examined p21 mRNA expression in intestinal mucosa of these mice at the same timepoint. Baseline p21 mRNA expression was reduced by 4.7-fold in the un-irradiated mice treated with AZ31 (Fig. 5E). While 9 Gy TBI resulted in a 2.5-fold upregulation of p21 expression in the vehicle treated mouse, AZ31 treatment reduced radiation-induced p21 mRNA to only 1.3-fold above baseline. Conversely, AZD6738 increased radiation-induced p21 mRNA by 3.3-fold.

### Impact of ATM and ATR kinase inhibition on intestinal crypts in *Cdkn1a(p21* ^*CIP/WAF1*^)*−/−* mice

**Figure 6:**
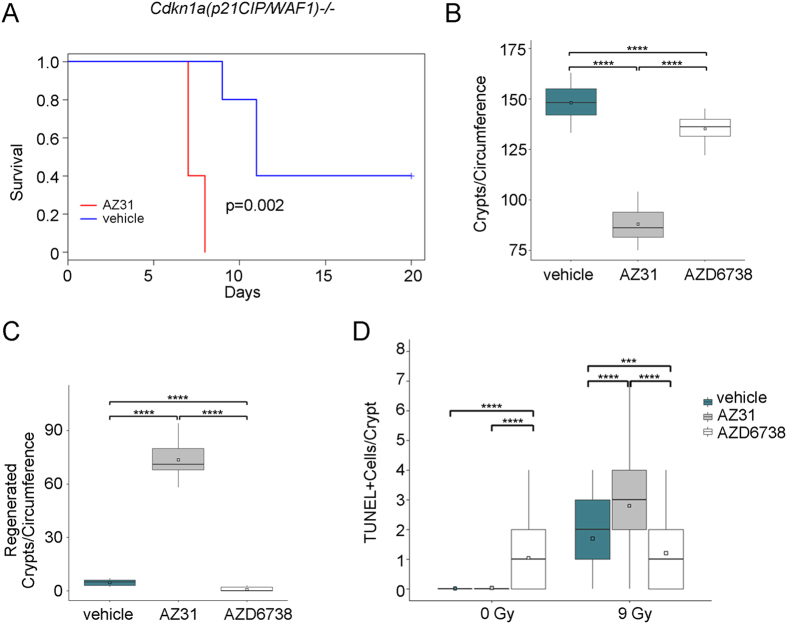
Impact of ATM and ATR kinase inhibition on survival and intestinal crypt health in *Cdkn1a(p21*^*CIP/WAF1*^)*−/−* mice. (**A**) Kaplan-Meier survival curves for *Cdkn1a(p21*^*CIP/WAF1*^)*−/−* mice treated with vehicle or 100 mg/kg AZ31 2 h prior to 9 Gy TBI. N = 5 mice per cohort. (**B,C**) *Cdkn1a(p21*^*CIP/WAF1*^)*−/−* mice received vehicle, 100 mg/kg AZ31, or 75 mg/kg AZD6738 2 h prior to 9 Gy TBI. Small intestine tissues were harvested at 7 days after TBI. The total number of crypts per circumference of small intestine (**B**) and the number of regenerated crypts per circumference of small intestine (**C**) were enumerated. Box and whisker plots depict 12–16 total circumferences (n = 12–16), with 6–8 circumferences from each of 2 mice. ****p < 0.0001. (**D**) *Cdkn1a(p21*^*CIP/WAF1*^)*−/−* mice received vehicle, 100 mg/kg AZ31, or 75 mg/kg AZD6738 2 h prior to 9 Gy TBI. Small intestine tissues were harvested at 4 h after TBI. For un-irradiated mice, tissues were harvested at 6 h after inhibitor dosing, equivalent to 4 h after TBI. The number of TUNEL positive cells per small intestine crypt was enumerated. Box and whisker plots depict counts from a total of 200 crypts (n = 200), with 100 crypts from each of 2 mice. ***p < 0.001, ****p < 0.0001.

*Cdkn1a(p21*^*CIP/WAF1*^)*−/−* mice exhibit increased sensitivity to TBI^38^. To determine whether the GI effects observed in wild-type mice treated with AZ31 prior to 9 Gy TBI is entirely dependent on ablation of the p21-dependent G1 cell cycle checkpoint by AZ31, we treated *Cdkn1a(p21*^*CIP/WAF1*^)*−/−* mice with vehicle or 100 mg/kg AZ31 2 h prior to 9 Gy TBI. Mice were euthanized when moribund. AZ31 reduced median survival of *Cdkn1a(p21*^*CIP/WAF1*^)*−/−* mice after 9 Gy TBI from 11 to 7 days (p = 0.002) (Fig. 6A). Of the five mice treated with AZ31, three mice became moribund at day 7 and two at day 8. Of the mice treated with vehicle, one mouse became moribund at day 9 and two at day 11, while the remaining two mice survived until end of study.

Since time to onset of moribund in the *Cdkn1a(p21*^*CIP/WAF1*^)*−/−* mice treated with AZ31 was 7 days after TBI, we examined the small intestine at this timepoint following treatment with vehicle, 100 mg/kg AZ31, or 75 mg/kg AZD6738 2 h prior to 9 Gy TBI. While AZD6738 treatment resulted in a modest reduction in the number of crypts per circumference of small intestine after TBI (mean 148 for vehicle vs. 135 for AZ31, p < 0.0001), AZ31 treatment was associated with significant crypt loss (mean 148 for vehicle vs. 87.9 for AZ31, p < 0.0001) (Fig. 6B). ATM inhibition prior to TBI also resulted in a significant increase in the number of regenerated crypts at this timepoint (mean 4.58 for vehicle vs. 73.5 for AZ31, p < 0.0001) (Fig. 6C).

We also examined cell death in the intestinal crypts of *Cdkn1a(p21*^*CIP/WAF1*^)*−/−* mice treated with vehicle, 100 mg/kg AZ31, or 75 mg/kg AZD6738 2 h prior to 0 Gy or 9 Gy. We performed the TUNEL assay for fragmented DNA and enumerated the number of positive cells per crypt at 4 h after TBI. Treatment with AZD6738 induced fragmented DNA in intestinal crypts of un-irradiated mice (mean 0.02 for vehicle vs. 1.05 for AZD6738, p < 0.0001) (Fig. 6D). The number of cells with fragmented DNA following ATR inhibition prior to TBI was reduced compared to TBI alone (mean 1.70 for vehicle vs. 1.21 for AZ6738, p < 001). However, ATM inhibition prior to TBI increased the number of intestinal crypt cells with fragmented DNA at this timepoint (mean 1.70 for vehicle vs. 2.70 for AZ31, p < 0001).

## Discussion

ATM kinase activity is essential in intestinal crypts to prevent radiation-induced GI syndrome. We have explored the physiological consequences of ATM kinase inhibition using AZ31^40^ and ATR kinase inhibition using AZD6738^26^ prior to TBI in mice. ATM kinase inhibition using AZ31 prior to 9 or 9.25 Gy TBI reduced median time to moribund in mice to 8 days. This time to moribund, of between 7 and 12 days, is consistent with radiation-induced GI syndrome^2^,^3^. Furthermore, the striking pathology associated with ATM kinase inhibition prior to TBI in moribund mice was increased crypt loss within the intestine epithelium. ATM kinase inhibition prior to 9 Gy TBI clearly increased intestinal crypt cell death at 24 h, intestinal crypt loss from 24 h to 96 h and crypt regeneration by 96 h. This is significant radiosensitization by ATM kinase inhibitor AZ31 as intestinal crypt regeneration and crypt loss have not been seen previously in C57Bl6 mice exposed to less than 12 Gy TBI in the Hillman Cancer Center Animal Facility^35^,^42^,^44^. ATM kinase inhibition, however, did not potentiate radiation-induced pathology in brain, esophagus, femur, heart, kidney, liver, lung, spleen, sternum or stomach. It is not clear how ATM kinase activity is essential in intestinal crypt cells, but not these other cell types after TBI.

ATM kinase activity is essential for the upregulation of p21 and the G1 cell cycle checkpoint in intestinal crypt cells after TBI. ATM inhibition blocked the expression of p21 mRNA in undamaged crypt cells and reduced upregulation of p21 mRNA and protein in crypt cells after TBI. ATM kinase inhibition blocked G1 arrest increasing the number of cells in S phase in crypts in wild-type, but not *Cdkn1a(p21*^*CIP/WAF1*^)*−/−* mice after TBI. Our data show that the radiation-induced, p21-dependent G1 cell cycle checkpoint^36^,^37^ is entirely ATM kinase-dependent in intestinal crypt cells. Nevertheless, ATM kinase inhibition reduced median survival of *Cdkn1a(p21*^*CIP/WAF1*^)*−/−* mice after 9 Gy TBI from 11 to 7 days. This is a striking finding as p21-dependent, G1 cell cycle arrest in intestinal crypt stem cells is believed to prevent GI syndrome after irradiation^3^,^35^,^36^,^37^,^38^,^39^.

One potential explanation for the radiosensitization of *Cdkn1a(p21*^*CIP/WAF1*^)*−/−* mice by ATM kinase inhibitor is that the p21-dependent G1 cell cycle checkpoint may promote ATM kinase-independent DNA repair, for example non-homologous end joining (NHEJ) which is the principal mechanism of DSB repair in G1 phase cells, while concurrently decreasing ATM kinase-dependent DNA repair, for example homologous recombination repair (HRR), a mechanism of DSB repair that is generally restricted to S and G2 phase cells. This potential explanation is supported by the recent finding that pharmacologic G1 cell cycle arrest with a CDK4/6 inhibitor increases NHEJ and decreases HRR^45^. However, arguing against this potential explanation, p21 has been shown to promote the repair of DSBs by HRR and sister chromatid exchange (SCE), a cytological manifestation of the HRR of stalled and collapsed replication forks, is reduced in *Cdkn1a(p21*^*CIP/WAF1*^)*−/−* cells treated with clastogens^46^.

A second potential explanation for the radiosensitization of *Cdkn1a(p21*^*CIP/WAF1*^)*−/−* mice by ATM kinase inhibitor is that ATM kinase inhibition may disrupt a p53-dependent, p21-independent mechanism(s) of radioprotection. Consistent with this potential explanation, *Trp53−/−* mice are more sensitive to TBI than *Cdkn1a(p21*^*CIP/WAF1*^)*−/−* mice and this has been attributed, at least in part, to disruption of p53-, PUMA-dependent apoptosis and an associated accelerated crypt regeneration and crypt loss^38^. Apoptosis in intestinal crypts is reduced in *PUMA−/−* mice^38^ and we show that apoptosis is also reduced by ATM kinase inhibition in wild-type mice after TBI. Thus, p53-, PUMA-dependent apoptosis contributes to radioprotection in the GI tract this mechanism may be disrupted by ATM kinase inhibition. However, PUMA deficiency does not rescue delayed nonapoptotic crypt death or shortened survival in *Cdkn1a(p21*^*CIP/WAF1*^)*−/−*38. Furthermore, *Atm−/−* mice are more radiosenstitive than *Trp53−/− mice* and *Atm−/−* and *Atm−/−, Trp53−/−* double knockout mice have been shown to exhibit similar radiosensitivity in the GI tract after TBI^47^. It is therefore likely that that additional ATM kinase-dependent and p53-independent mechanisms radioprotect the GI tract.

A third and our favored explanation for the radiosensitization of *Cdkn1a(p21*^*CIP/WAF1*^)*−/−* mice by ATM kinase inhibitor AZ31 is that ATM kinase inhibition may directly block the repair of DSBs induced by TBI. The rapid kinetics of DNA fragmentation at 4 h after TBI in *Cdkn1a(p21*^*CIP/WAF1*^)*−/−* mice are consistent with such a model. We have previously shown that ATM kinase inhibition but not ATM protein disruption blocks sister chromatid exchange in cultured cells^48^. Furthermore, while *Atm−/−* knockout mice are viable^12^,^13^, expression of ATM kinase-inactive in knockin mouse models causes early embryonic lethality^14^,^15^. These and the data presented here are consistent with a model in which ATM kinase-inhibited is dominant inhibitory over a DSB repair mechanism and we contend that ATM kinase inhibition may potentiate the cytotoxic effects of radiation in the GI tract in large part by poisoning DSB repair.

ATR kinase inhibition using AZD6738 prior to 9 or 9.25 Gy TBI did not significantly impact median time to moribund in mice. ATR inhibition increased apoptotic death in crypt cells in wild-type and *Cdkn1a(p21*^*CIP/WAF1*^)*−/−* mice that were not irradiated. This is presumably associated with an essential role for ATR in rapidly proliferating cells and is consistent with reports that ATR kinase inhibitors induce ATM kinase activity in the absence of exogenous insult^23^,^49^. ATR inhibition increased death in crypt cells in *wild-type* mice and this was associated with a reduced number of crypts after TBI, but the effect of ATR kinase inhibitors was considerably less than that of ATM kinase inhibitors.

Recent reports show that ATR is required at the mitochondrial membrane to block apoptosis^50^,^51^, and it is therefore possible that ATR kinase inhibitors may radioprotect certain cell types after TBI. ATR kinase inhibition increased upregulation of p21 after TBI. Thus, ATR inhibition may potentiate G1 cell cycle arrest in crypt cells after TBI. ATR inhibition also decreased cell death in crypts in *Cdkn1a(p21*^*CIP/WAF1*^)*−/−* mice at 4 h after TBI. This may reflect some slowing of DNA replication and a corresponding delay in DNA damage and cell death induction, perhaps via the activation of ATM kinase-dependent intra-S phase checkpoint. It may also reflect the preferential induction of HRR in the absence of the G1 cell cycle checkpoint described above, which may be preferentially induced further by the activation of ATM kinase activity by ATR inhibitors. *In toto*, while our data suggest that ATR kinase inhibitors may radioprotect intestinal crypts cells at early timepoints, at this time we can only conclude that ATR kinase inhibitors do not radiosensitize intestinal crypt cells following TBI.

The significance of this work is that ATM kinase inhibitors, but not ATR kinase inhibitors, promote GI syndrome after TBI and this should inform the design of clinical trials with ATM kinase inhibitors. Clinical trials of ATM kinase inhibitors with abdominal radiation or genotoxic chemotherapy that has dose-limiting toxicity in intestinal crypts may be less likely to provide sufficient benefit versus risks, while ATR kinase inhibitors, which are currently in clinical trials, may have utility in these contexts.

## Methods

### Drugs and reagents

The ATM kinase inhibitor, AZ31, and the ATR kinase inhibitor, AZD6738 were provided by AstraZeneca. AZ31 and AZD6738 were dissolved in DMSO at concentrations of 100 mg/mL and 75 mg/mL, respectively. AZ31 was diluted 1:10 in Captisol (30% w/v in dH_2_O) to yield 10 mg/mL AZ31 in 10% DMSO, 90% Captisol. AZD6738 was diluted 1:5 in propylene glycol. An equivalent volume of dH_2_O was added to yield 7.5 mg/mL AZD6738 in 10% DMSO, 40% propylene glycol, 50% dH_2_O. Bromodeoxyuridine (BrdU, Simga-Aldrich) was dissolved in DMSO at a concentration of 33.33 mg/mL, and diluted in saline (0.9% NaCl) to yield 10 mg/mL BrdU in 30% DMSO, 70% saline.

### Mice and treatments

Experimental procedures were approved by the University of Pittsburgh Animal Care and Use Committee and all methods were performed in accordance with the relevant guidelines and regulations. C57BL6/J mice were purchased from Taconic Biosciences. *Cdkn1a(p21*^*CIP/WAF1*^)*−/−* C57BL6/J mice were bred from previously established breeders^38^. Eight to eleven week old mice were used. Animals received 100 mg/kg AZ31 and/or 75 mg/kg AZD6738 or vehicle by oral gavage 2 h prior to TBI. Mice were irradiated in a ^137^Cs irradiator at 67 cGy/min. Mice were injected intraperitoneally with 100 mg/kg BrdU 2 h prior to tissue harvest. The dosing volume for all chemicals was 10 mL/kg.

### Pharmacokinetics of AZ31 and AZD6738

Plasma, thymus, and small intestine scrapings were collected at 2 h after dosing. Plasma samples and tissue homogenate were analyzed with an LC-MS/MS assay validated according to US FDA guidance for bioanalytical method validation, which was accurate (−10.7 to 7.6%), precise (<11.1% CV), and linear from 10–5,000 ng/mL (submitted for publication).

### Immunohistochemistry

Deparaffinized, rehydrated sections were treated with 3% hydrogen peroxide to quench endogenous peroxidase. For BrdU staining, sections were treated with 20 ug/mL proteinase K for 20 min at 37 °C. For BrdU staining, antigen retrieval was performed in 2 M HCl at room temperature for 1 h. For p21 and Cleaved Caspase-3 staining, heat-based antigen retrieval was performed. Sections were blocked in 20% goat serum for 1 h. Primary antibody incubation was performed overnight at 4 °C with BrdU (Life Technologies, B35138; 1:20 in 10% goat serum), p21 (Santa Cruz Biotechnology, sc-6246: 1:100 in 10% goat serum) or Cleaved Caspase-3 Asp175 (Cell Signaling Technology, #9664; 1:500 in 2% goat serum). Secondary antibody incubation was performed at room temperature for 1 h with biotin conjugated goat anti-mouse IgG (Pierce, #31802; 1:100) for BrdU and p21, or PowerVision Poly-HRP anti-rabbit IgG (Leica, PV6119) for Cleaved Caspase-3. For BrdU and p21 staining, signal was amplified using the VECTASTAIN Elite ABC Kit (Vector Labs). Staining was developed using the DAB Peroxidase Substrate Kit (Vector Labs). TUNEL was performed using the ApopTag Plus Peroxidase *In Situ* Apoptosis Kit (EMD Millipore).

### qRT-PCR for p21 expression

Small intestine mucosal scrapings were collected from mice 24 h after 9 Gy TBI. Mucosa scrapings were homogenized in 700 uL RNA Lysis buffer and total RNA was isolated. cDNA was generated and quantitative real-time PCR was performed as previously^38^. Expression of p21 was normalized to GAPDH, and fold change in p21 expression relative to vehicle control was determined.

### Statistical Methods

Differences in overall survival times were compared using log-rank tests. Median survival times were estimated using the Kaplan-Meier method and Kaplan-Meier survival curves were generated. Treatment group differences in number of cells per crypt and number of crypts per circumference were assessed using generalized estimating equations (GEE) for either poisson or negative binomial regression models, as appropriate, with an exchangeable correlation matrix. P-values are 2-sided and are adjusted for performing multiple comparisons between each pair of treatment arms by the Tukey-Kramer method. Adjusted p-values < 0.05 are considered statistically significant. Analyses were performed with SAS 9.4 (SAS Institute, Cary NC) and R 3.1.1.

## Additional Information

**How to cite this article**: Vendetti, F. P. *et al*. Pharmacologic ATM but not ATR kinase inhibition abrogates p21-dependent G1 arrest and promotes gastrointestinal syndrome after total body irradiation. *Sci. Rep.* **7**, 41892; doi: 10.1038/srep41892 (2017).

**Publisher's note:** Springer Nature remains neutral with regard to jurisdictional claims in published maps and institutional affiliations.
